# Interplay between Cartilage and Subchondral Bone Contributing to Pathogenesis of Osteoarthritis

**DOI:** 10.3390/ijms141019805

**Published:** 2013-09-30

**Authors:** Ashish R. Sharma, Supriya Jagga, Sang-Soo Lee, Ju-Suk Nam

**Affiliations:** Infectious Diseases Medical Research Center/Institute for Skeletal Aging, College of Medicine, Hallym University, Chuncheon 200702, Korea; E-Mails: boneresearch@hallym.ac.kr (A.R.S.); supriya.jagga@gmail.com (S.J.); totalhip@hallym.ac.kr (S.-S.L.)

**Keywords:** osteoarthritis, wingless-type (WNT), bone morphogenic protein (BMP), mitogen-activated protein kinases (MAPKs), cartilage, subchondral bone

## Abstract

Osteoarthritis (OA) is a common debilitating joint disorder, affecting large sections of the population with significant disability and impaired quality of life. During OA, functional units of joints comprising cartilage and subchondral bone undergo uncontrolled catabolic and anabolic remodeling processes to adapt to local biochemical and biological signals. Changes in cartilage and subchondral bone are not merely secondary manifestations of OA but are active components of the disease, contributing to its severity. Increased vascularization and formation of microcracks in joints during OA have suggested the facilitation of molecules from cartilage to bone and *vice versa*. Observations from recent studies support the view that both cartilage and subchondral bone can communicate with each other through regulation of signaling pathways for joint homeostasis under pathological conditions. In this review we have tried to summarize the current knowledge on the major signaling pathways that could control the cartilage-bone biochemical unit in joints and participate in intercellular communication between cartilage and subchondral bone during the process of OA. An understanding of molecular communication that regulates the functional behavior of chondrocytes and osteoblasts in both physiological and pathological conditions may lead to development of more effective strategies for treating OA patients.

## 1. Introduction

Osteoarthritis (OA) is a degenerative joint disease characterized by progressive degeneration of articular cartilage, osteophyte formation, and subsequent joint space narrowing [1]. The ever growing prevalence and incidence of OA with age makes this condition a major healthcare problem affecting millions of people worldwide [2]. The entire race is affected and the disease is not confined to any particular geographical area. After the age of 50, the increase in incidence of severe OA is exponential [3]. Frequently affected sites include hands, knees, hips, and spine. The symptoms are often associated with significant functional impairment along with signs and symptoms of inflammation, stiffness, and loss of mobility [4]. The etiology of OA is multi-factorial and can be broadly divided into non-genetic and genetic factors. Non-genetic factors may include: age, gender, obesity, inactive lifestyle, joint injury and occupation (occupation related to bending of knees and heavy lifting) whereas genetic factors may be hereditary and altered gene expression pattern of cartilage and subchondral bone tissues. Epidemiological and clinical data support the view that OA is primarily a mechanically induced disease with genetic or acquired factors further contributing to its severity [5].

Anatomical progression of OA can be represented by structural alteration in articular cartilage leading to narrowing of the joint space. The articular surface plays an indispensable role in load transfer across the joint and there is enough evidence that conditions leading to increased load transfer and/or altered patterns of load distribution can accelerate the instigation and progression of OA [6]. Therefore, OA was initially treated as a disease of the cartilage. However, growing evidence suggests OA as an organ level failure, involving not only cartilage, bone marrow and bone, but also menisci, ligaments, muscles and neural tissues [7]. Since observable structural differences in articular cartilage and subchondral bone has been attributed to the progression of OA, an increasing number of studies are focused on their participatory role and function as targets for developing new therapeutics for the treatment of OA [8–11]. However, it remains unclear whether bone changes occur before or after cartilage changes related with clinical symptoms [12,13] and support of both chronologies has been given in the literature. Nevertheless, it is obvious that changes in both tissues occur before clinical symptoms manifest themselves, and can therefore be considered as a cause of the abnormal state leading to OA.

In joints, the close physical relationship between the cartilage and subchondral bone has introduced the concept of biochemical and molecular crosstalk across the affected region. In knee OA, chondrocytes can respond to direct biomechanical perturbation by modulating synthetic activity or by increasing the production of inflammatory cytokines. On the other hand, underlying subchondral bone is affected by altered bone remodeling processes leading to sclerosis. The biomechanical alteration between the articular cartilage and the underlying bone raises the question of molecular crosstalk between chondrocytes and bone cells (osteoblasts, osteoclasts and osteocytes) and if it exists, the extent to which it may contribute to the progression of OA.

Therefore, in the present review, we discuss the various signaling mechanisms that may allow reciprocal communication between articular cartilage and subchondral bone, and how their modulation during pathogenesis contributes to progression of bone diseases like OA.

## 2. Onset and Progression of OA

**Figure 1:**
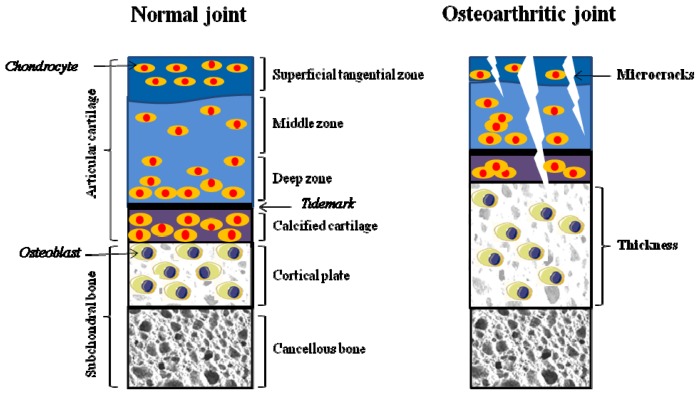
A schematic diagram demonstrating the anatomy of articular cartilage and subchondral bone in normal and osteoarthritis (OA) joints. Normal articular cartilage is divided into superficial tangential zone, middle zone, deep zone, and calcified cartilage zone. These zones consist of a small number of chondrocytes trapped in collagen matrix. Calcified cartilage is separated by a tidemark from the deep zone, and rests directly on subchondral bone. Subchondral bone beneath the articular cartilage is organized into two layers: cortical plate and cancellous bone. Subchondral bone helps to maintain the integrity of the overlying articular cartilage. Alteration in OA joint is represented by collagen matrix disruption in articular cartilage and thickening of subchondral bone. Fissuring and flanking in articular cartilage induces vascularization of cartilage, leading to exposure of subchondral bone to external surface. Microcracks in the subchondral bone contribute to reactivation and upward shifting of the tidemark (demarcation line), representing thin articular cartilage with thick subchondral cortical plate. Subchondral sclerosis is a hall mark of progressive OA. (Figure produced using Servier Medical Art [17]).

A diarthrodial joint is an intricate structure comprising various connective tissues including articular cartilage, synovial membrane, subchondral bone, ligaments and sometimes menisci [14]. Broadly, articular cartilage consists of four zones; the superficial, middle, deep and calcified zone (Figure 1). Superficial articular cartilage is composed of a highly structured network of extracellular matrix proteins, proteoglycans, collagen, and non-collagenous proteins, and maintains a high water content. Middle layer contains randomly oriented collagen fibers with larger chondrocytes. Deep stratum is arranged in vertical columns separated by collagenous fibrils. The last layer of articular cartilage is composed of calcified cartilage with partial mineralization and hypertrophic chondrocytes. The calcified zone is separated from the deep zone by a thick bundle of collagen fibrils termed the tidemark [15]. Subchondral bone is the zone of epiphyseal bone just underneath the articular cartilage, and includes the subchondral bone plate and the underlying trabecular bone. The subchondral bone plate includes the deepest area of the articular cartilage, which is the calcified cartilage and a thin cortical bone layer [16] (Figure 1).

### 2.1. Alteration in Cartilage

During their entire lifespan, articular chondrocytes along with subchondral bone cells perceive constant acute or chronic stress and react accordingly. Biomechanical stress is necessary for the maintenance of joint homeostasis, as there is rapid loss of proteoglycans in joints that are immobilised or in disuse [18]. However during OA, homeostatic or reparative processes cannot sufficiently compensate for destructive mechanisms, resulting in structural damage and clinical symptoms [19]. The consensus from *in vitro* studies indicates that mechanical loading representing injurious static compression can stimulate the depletion of proteoglycans and damage the collagen network. These events decrease the synthesis of cartilage matrix proteins, whereas dynamic compression increases matrix synthetic activity [20]. Function of articular cartilage is load bearing, and the low water content of cartilage allows it to perform under compressive loads without failure [21]. However, cartilage cannot withstand high tension or shear at the edges of the joint contact regions for a long time and predisposes the cartilage to splitting or fibrillation. OA cartilage is characterized by an initial loss of proteoglycan from the upper zone followed by the degradation of the collagen network. During the progressive stages, the collective alterations in the molecular composition and organization of the cartilage matrix leads to deterioration in the material properties and structural integrity of the articular surface and underlying hyaline cartilage [22]. Chondrocytes represent the only cell type residing in the adult cartilage matrix, possessing a low metabolic activity, surviving under hypoxic conditions (<5% pO_2_ compared to >12% pO_2_ in arterial blood) and in the absence of a vascular supply [23]. Chondrocytes possess receptors for responding to biomechanical perturbation in the surrounding cartilage matrix, as well as intrinsic and extrinsic growth factors, cytokines and other inflammatory mediators [24]. Several integrins, which serve as receptors for fibronectin (FN) and type II collagen (COL2) fragments, on activation can stimulate the production of matrix-degrading proteinases and inflammatory cytokines and chemokines in chondrocytes [25]. Significant phenotypic modulation of chondrocytes by increased synthesis of FN, COL2 and aggrecan (AGG), just after onset of disease suggests that articular chondrocytes try to repair the damaged matrix. Nevertheless, this repair process eventually appears to fail, leading to irreversible cartilage degeneration [26,27]. Cartilage degradation is often accompanied by the elevated presence of some key biochemical markers during onset and progression of OA (Table 1).

### 2.2. Alteration in Subchondral Bone

Along with progressive loss of articular cartilage, OA is characterized by increased subchondral bone sclerosis with thickening of cortical plate, extensive remodeling of the trabeculae, formation of new bone at the joints margins (osteophytes) and the development of subchondral bone cysts [38,39]. Like cartilage, an increase in expression level of certain genes in bone is regarded as biochemical markers for OA (Table 1). The term subchondral has been defined to include both subchondral cortical plate and the cancellous bone. Anatomically, the subchondral cortical plate is not very porous or vascular in nature and represents corticalized bone similar to other skeletal locations. While, subchondral cancellous bone is more porous, it has a lower volume and density and stiffness than the cortical plate [40]. Both the subchondral cortical plate and cancellous bone shows distinct difference in their behavior during progression of OA and hence must be regarded as separate units to understand the joint deformation events during OA [41–43]. During progression of OA, subchondral bone turnover appears to be 20-fold increased compared to normal bone turnover [44]. It has been reported that subchondral bone explants from OA patients, secrete high levels of alkaline phosphatase (ALP), osteocalcin, osteopontin, IL-6, IL-8, and progressive ankylosis protein homolog (ANKH), urokinase plasminogen activator (uPA), prostaglandin and insulin growthfactor-1 (IGF-1) compared to normal bone explants [45]. Moreover, subchondral bone osteoblasts from OA patients have been shown to express higher level of ALP, osteopontin and, osteocalcin mRNA and type 1 collagen protein, and growth factors like IGF-1, IGF-2 and (transforming growth factor-β) TGF-β than normal subchondral bone osteoblasts [46,47]. These secreted biochemical factors that contribute to bone formation, suggesting an enhanced bone anabolic activity of subchondral bone osteoblasts, exemplified by formation of osteophytes. However, the bone forming activity of subchondral bone is not necessarily accompanied by equivalent mineralization. Increasing evidence suggests that unmineralized immature new bone formation leads to abundant osteoids in the subchondral bone (both at the level of the cortical plate and at the level of the trabecular bone) resulting in the opposite effect on tissue properties [48].

Although both cartilage and bone are affected during the progression of OA, the precise mechanisms that lead to increased joint stiffness in OA remains to be elucidated. There have been numerous research efforts on understanding the pathological association of articular cartilage and subchondral bone to OA but still some questions persist. Key questions can be whether changes in the properties of one side can alter the load bearing capability of the other and *vice versa* and the extent to which the physical and molecular interaction between them may affect and contribute to progression of OA.

## 3. Cross Talk between Articular Cartilage and Subchondral Bone

The close physical association between subchondral bone and cartilage led to the concept of biochemical and molecular crostalk across this region during OA. As articular chondrocytes are physically separated from underlying subchondral bone by calcified cartilage [49], the possibility of any paracrine regulation remained questionable. The extent of matrix mineralization in adult bone and cartilage even reduces the chances of any functional interaction. However, the recently reported presence of increased vascularization and development of microcracks in the bone matrix, strongly suggest that mediators secreted from chondrocytes and subchondral bone cells could directly interact through these channels [49,50]. It has been observed that products derived from cartilage or subchondral bone are secreted into the joint space, from where they can gain access to cartilage or bone through the synovial fluid [51]. Studies in animals suggest that nutrients from medullary cavity in bone may nourish cartilage via channels that connect bone with cartilage as well as blood vessels [52,53]. Even in a bovine explants experiment, subchondral bone significantly influenced the chondrocyte survival during explant culture [54]. Moreover, in OA, chondrocyte-secreted regulatory factors within the degraded cartilage may play a role in osteoclastogenesis, and thus contribute to subchondral bone loss [55]. Cartilage hypertrophy during endochondral ossification seems to be the result of signals derived from various cells like osteoblasts and haemapoietic cells [56,57]. On the contrary, it was demonstrated that the signals from chick hypertrophic cartilage chondrocytes stimulates osteoblast differentiation and subsequent bone matrix deposition [58].

Taken together, all these evidences indicate that subchondral bone and cartilage are dynamic load-bearing structures; they: (1) are capable of bearing the load; (2) change metabolism; and (3) can even respond to load by varying its biomechanical characteristics. Therefore, to have an insight into the understanding of possible cellular and molecular interaction leading to the progression of OA, we classified various factors under the following subheadings and discussed in each section.

### 3.1. Biological Factors

Even though at macroscopic observational scale, there is obvious coupling of bone and cartilage, more research is needed to understand the cellular and molecular interaction between osteoblasts and chondrocytes, to elucidate and identify the various factors affecting initiation and progression of OA.

OA is not regarded as a classical immunological arthropathy, because it lacks the absence of immune cells in the synovial fluid and systemic manifestations of inflammation. However, signs and symptoms of inflammation along with joint pain, swelling and stiffness are clearly evident during its progression. Though there is debate regarding the essential role of synovial inflammation in OA, synovitis involving infiltration of activated B cells and T lymphocytes along with overexpression of proinflammatory mediators has been reported during early and late OA [59]. Proinflammatory mediators present in synovial fluid are likely to contribute to catabolic activities of chondrocytes leading to remodeling of the cartilage extracellular matrix [60]. Besides, an increased vascularity deep into articular cartilage, under stimulation of angiogenic factors such as vascular endothelial growth factor (VEGF) in synovial fluid, is also found in patients with OA [61]. Evidences from *in vivo* and *in vitro* studies have clearly reported that a number of chemokines and cytokines present in synovial fluid are enough to activate chondrocytes to increase the synthesis of matrix molecules but at the same time contributes to their own destruction by synthesizing proinflammatory cytokines and proteases. During OA, chondrocytes have been found to secrete IL-1, IL1β converting enzyme (caspase-1) and type 1 IL-1 receptor. Concentration at which IL-1 is synthesized by chondrocytes is capable of inducing the expression of matrix metalloproteinases (MMPs), aggrecanases, adisintegrin and metalloproteinase with thrombospondin motifs (ADMATs) and other catabolic genes in regions of matrix depletion in OA cartilage [22]. Alterations in the remodeling process of cartilage lead to the loss of extra cellular matrix components and structure, affecting the characteristic phenotype of the articular chondrocytes. Under these conditions, chondrocytes are stimulated to express molecules that are associated with chondrocyte hypertrophy and terminal differentiation, like VEGF, runt-related transcription factor 2 (RUNX2) and MMP-13. These events lead to a shift towards increased hypertrophy, accompanied by calcification of the extracellular matrix around chondrocytes, and might promote thinning of the soft articular surface. Secretion of angiogenic factors such as VGEF, increase vascularity within the deep layers of articular cartilage facilitating molecular transport by diffusion of molecules through the calcified tissues from and into the articular cartilage and the subchondral bone. Recently, chemokines, cytokines and proteases secreted from chondrocytes have been implicated to alter biochemical and functional abilities of subchondral bone osteoblasts. For example, IL-6 in combination with other cytokines like IL1β can switch osteoblasts from a normal phenotype to a sclerotic phenotype [62]. Furthermore, knockout of *ADMATs5* in mice reduced the subchondral bone effects of joint destabilization and protected the overlying cartilage from degeneration [63]. Enhanced secretion of insulin growth factor, TGF-β and prostagaldin E_2_ has been detected from deteriorating cartilage at the protein level [64]. All these can potentiate and stimulate the process of bone remodeling, altering the physiology of subchondral bone. Chondrocytes undergoing destruction are also known to secrete increased amounts of osteoclastogenesis inducing factor, receptor activator of nuclear factor κ-B ligand (RANKL). Elevated expression of RANKL has been shown to associate with an increased turnover of subchondral bone in the early stages of OA [65,66].

Apart from the stimulatory role of chondrocytes on subchondral bone, increasing evidence for a number of subchondral bone factors that are involved in both tissue remodeling and the modulation of cartilage catabolism has been demonstrated. *In vitro* experiments have shown that OA subchondral bone osteoblasts decrease the cartilage specific phenotype [glycosaminoglycan (GAG), AGG, COL2] upon co-culture with articular chondrocytes [67]. Also, subchondral bone osteoblasts cultured from OA patients have shown increased proteoglycan degradation in cartilage compared to healthy controls [51]. Increased MMP-2 production by subchondral bone osteoblasts may mediate proteoglycan degradation in cartilage [68]. A decreased osteoprotegerin:RANKL ratio has been observed in animal models of OA [69]. Osteoblasts are the major source of RANKL [70], and thus may contribute to elevated osteoclastogenesis observed during progression of OA. Recently, a 50% decrease in cartilage pathology score, as measured by the golden standard Mankin score, was reported on extensive inhibition of bone resorption, implicating the importance of osteoclast function and bone turnover in the pathogenesis of OA [71,72]. Though osteoclasts have been known to play important roles in the degradation of cartilage in rheumatoid arthritis [73,74], their role in the pathogenesis of OA still needs more attention.

Hepatocyte growth factor (HGF) may also play an important role in the interaction between cartilage and subchondral bone. An increased expression and production of hepatocyte growth factors (HGF) is detected in human subchondral bone osteoblasts, whereas only HGF protein, not mRNA, has been detected in human articular cartilage from OA patients [72,75,76]. It is detectable only in the intermediate and deep zone of human OA cartilage thus suggesting that only subchondral bone is able to secrete them, which then diffuses to the region of cartilage [76]. HGF is known to induce the expression of collagenase-3 or MMP-13 in human OA cartilage and is present in the intermediate and deep layers of articular cartilage [77], similar to the site of HGF.

Recently the presence of sphingosine 1-phosphate (S1P) has been reported in the synovial fluid of OA patients. Synovial tissue is a potential source of S1P. Human cartilage chondrocytes express S1P receptors on their surfaces. S1P is involved in the regulation of cyclooxegenase-2 (COX-2), VEGF and in expression of inducible nitric oxide synthase (iNOS), MMP-13, and ADAMTS-4 in cartilage chondrocytes [78,79]. VEGF involvement is suggested in osteophyte formation by regulating angiogenesis [79]. Thus the presence of S1P in synovial fluid can mediate the expression of catabolic proteases and VEGF in chondrocytes to promote angiogenesis at the osteochondral border resulting into osteophyte formation.

### 3.2. Signaling Pathways

The maintenance of joint function is dependent on molecular regulators of the bone-cartilage biomechanical unit. Homeostasis of joints is critically dependent on the balance between various anabolic and catabolic signaling pathways [80]. Signaling mechanisms at joints are required to maintain the stable phenotype for articular cartilage and subchondral bone, sustained extracellular matrix (ECM) synthesis, balancing of bone remodeling process, efficient breakdown and clearance of macromolecules and dead cells, and functional and molecular adaptations to experienced mechanic loads. A number of signaling pathways have been reported to co-exist in joints required to maintain this homeostasis and subsequent maintenance. However, an imbalance of the delicate equilibrium between them, due to a number of reasons as discussed in above sections, leads to a gradual deterioration of cartilage quality and thickening of the subchondral bone contributing to the progression of OA.

In this section, we have tried to summarize a few of the signaling pathways that have been implicated in OA pathogenesis, and an understanding of which of these may contribute to the development of future therapeutics.

#### 3.2.1. WNT Signaling

The wingless-type (WNT) signaling pathway plays a vital role during early development, organogenesis and to maintain tissue homeostasis [81]. WNTs constitute a large family of cysteine-rich morphogens. As evidenced in mice and humans, there are a group of at least 19 structurally related glycoproteins which can transduce their signal through different intracellular cascades [82]. Classically, WNTs have been classified as “canonical” or “non-canonical”. On the basis of their ability to inhibit phosphorylation of β-catenin by GSK-3β and its subsequent degradation, they are termed as canonical (e.g., WNT 1, 3a, 8) whereas if they do not affect β-catenin levels, then are termed as non-canonical (e.g., WNT 4, 5a, 11).

WNT signaling plays an important role during skeletal patterning and is well associated with postnatal health and diseases [83]. Lately, WNT signaling has been reported to play an essential role in cartilage, bone and joint development [84]. Studies in mice show that canonical WNT signaling is necessary for the maintenance of mature articular cartilage phenotype, which is characterized by extended cell survival and absence of differentiation towards hypertrophy [85,86]. A surgically-induced model of OA in *Lrp5*^−/−^ (co-receptor for canonical WNTs) mice also showed increased apoptosis of chondrocytes, comparable to those documented in mice with conditional ablation of β-catenin [87]. Collectively, these observations imply that canonical WNT signaling acts as a survival signal, inhibiting apoptosis, for chondrocytes. Conversely, there are several reports which show that even over expression of WNT signaling is also deleterious to chondrocytes, leading to OA-like disease. Frizzled-related protein 3 (sFRP3, also known as Frzb) is a WNT antagonist [88] and polymorphisms in *FRZB* gene have been associated with OA [84]. Frzb-deficient mice do not develop spontaneous arthritis but are more susceptible to OA in induced models. More severe cartilage damage in *Frzb*^−/−^ mice compared to wild-type controls has been observed [89]. It has been suggested that an increase in WNT signaling induces the expression of catabolic factors such as MMPs and aggrecanase, contributing to cartilage damage [87]. Along with canonical WNT signaling, non-canonical WNT cascades are also implicated in cartilage biology [90]. During early development stages of mesenchymal cells to chondrocytes, non-canonical WNTs like WNT5a and WNT5b are required [91,92]. Dickkopf (DKK) 1 mediated inhibition of canonical WNT signaling demonstrates a non-canonical driven dedifferentiation process, particularly mediated by CaMKII and calcium signaling [90]. This study showed that a single WNT can simultaneously activate both canonical and non-canonical pathways to induce distinct and independent outcomes and with reciprocal regulation in chondrocytes. Involvement of both canonical and non-canonical cascades of WNT signaling during the proliferation and differentiation process of chondrocytes clearly implicates its importance in regulation of cartilage homeostasis.

Apart from chondrogenesis, WNT signaling is essential for bone development and its subsequent homeostasis [93]. Increased WNT signaling is capable of inducing sclerosis in bone [94]. In several experimental models, where WNT signaling has been activated either through knock down of WNT antagonists or receptors (*Frzb*^−/−^ or *Lrp5*^−/−^, respectively) or by overexpression of β-catenin, an increased bone formation is observed, leading to thicker and stiffer bones [85,87,89]. During OA, extensive remodeling of the existing subchondral cortical and trabecular bone leads to thickening of the subchondral end plate. Though the function of sclerotic subchondral bone in progression of OA is not clear, it has been suggested that it can contribute to deterioration of cartilage under altered mechanical load. Evidences from cartilage as well as underlying subchondral bone shows that the WNT signaling pathway has key modulatory roles in maintaining bone-cartilage biochemical unit. But, whether bone and cartilage can influence each other through modulation of WNT signaling is still a mystery. A vital signaling pathway among bone and cartilage is a suitable target for studying possible cross talk, and necessitates an understanding of its participatory role in altering bone-cartilage homeostasis during progression of OA. Indeed, a few studies collected so far point toward a possible mechanism that could explain and further direct us to understand the possible crosstalk in joint architecture mediated by WNT signaling.

**Figure 2:**
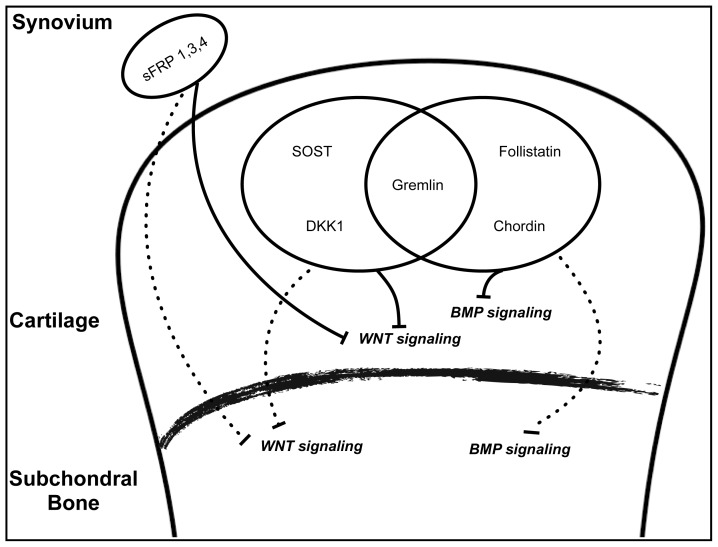
Schematic diagram representing possible interaction of antagonists of WNT and BMP signaling pathways during progression of OA.

Since WNT signaling is a complex event regulated by various secreted antagonists and agonists, cross talk and tight regulation may be expected to regulate WNT signaling among cartilage and subchondral bone for maintaining normal homeostasis. The presence of vessels and channels from subchondral bones into calcified and un-calcified cartilage, as observed in anatomical samples of human OA patients [52,95], further supports the possibility of diffusion of antagonists and agonists to either direction. Various studies have demonstrated differential expression of a range of antagonists and agonists during the progression of OA. Elevated expression of sclerostin (SOST), gremlin 1 [antagonists of WNT and bone morphogenic protein (BMP) signaling] and DKK1 has been reported in chondrocytes of articular cartilage of human OA samples [96,97]. Similarly, synovial cells like fibroblast and macrophages have also been found to sFRP1, 3 and 4 in synovium of patients with OA [98]. Inhibition of WNT signaling pathway by antagonists, both in cartilage and subchondral bone, may result into alteration in expressions of catabolic enzymes from chondrocytes and the remodeling process of subchondral bone. In support of this hypothesis, attenuation of DKK1 expression in knee joints by DKK1 antisense oligonucleotides promotes chondrocytes survival and protects against cartilage degradation and loss of subchondral bone mineral density (BMD) [99]. Hence, antagonists secreted from cartilage, bone or other synovial cells in joint spaces may become cross talk-effector molecules, modulating WNT signaling pathways for normal homeostasis to favor pathological conditions like arthritis (Figure 2). Moreover, overexpression of agonists of WNT signaling like WNT1-inducible signaling pathway protein 1 (WISP1) and WNT16 have also been reported from human cartilage explants on induction of cartilage injury and human OA synovium [100,101]. Secretion of agonists in joints may directly stimulate chondrocytes to secrete increased MMPs and aggrecanase to enhance deterioration of cartilage [101], whereas subchondral bone remodeling process may be altered to favor bone formation leading to osteophyte formation. A significant increase in osteocalcin (a marker for bone formation) level is detected in patients with OA, marking a shift of bone remodeling toward bone formation [102]. Observations from these studies are enough to speculate that normal homeostasis of cartilage and bone may be affected through altered expression of antagonists and agonists of WNT signaling. However, till date an ambiguity still exists about the source, timely involvement and precise mechanism of action of these factors on modulation of WNT signaling in joint biology.

In line with these observations, an additional role of WNT signaling in crosstalk of cartilage and subchondral bone biology has recently been reported. During late embryonic and postnatal development, the rate of trabecular bone formation is strongly dependent on the levels of β-catenin expressed by chondrocytes in the lower hypertrophic zone [103]. A level of β-catenin in hypertrophic chondrocytes is necessary for regulating the expression of RANKL to control osteoclast activity in subchondral growth plates. Thus, it may be expected that during the progression of OA, observed altered activity of WNT signaling in chondrocytes may affect the osteoclastic activity in subchondral bone growth plates leading to sclerosis or osteophyte formation at the edges of joints. Taken together, alteration of WNT signaling pathways in chondrocytes appears to modulate key regulatory factors for remodeling of subchondral bone, resulting in its aberrant behavior as observed in the case of OA. Similarly, altered WNT signaling in subchondral bone may modulate chondrogenic factors that are necessary for maintaining cartilage homeostasis.

Conclusively, WNTs have critical roles in the biology of chondrocytes, osteoblasts and osteoclasts along with cross talk among them, marking WNT signaling as a potential target for developing therapeutics. However, WNT signaling is more complex than it appears and thus careful strategies should be designed to weed out its regulatory roles in joint (patho) biology [90].

#### 3.2.2. TGF-β/BMP Signaling

TGF-β family of cytokines, including TGF-β and BMPs plays critical roles in embryonic development, adult tissue homeostasis and the pathogenesis of a variety of diseases. BMPs are highly conserved proteins having a multitude of functions, including regulation of extracellular matrix and bone remodeling in the skeletal system.

BMP signaling regulates the process of bone induction and is considered essential for endochondral bone formation. Being an intermediate stage during bone formation, BMPs are expected to be involved in all phases of chondreogensis [104]. BMP-2, -4 and -5 are required for chondrocytes proliferation and matrix synthesis [105]. BMP-2 has been shown to regulate the expression and activity of transcriptional factor sex determining region Y-box 9 (SOX-9), necessary for chondreogensis [106]. During endochondral bone formation, BMPs have been demonstrated to mediate these effects through canonical Smad molecules. Deletion of Smads (1, 5, and 8) in a cartilage specific manner in mice led to severe chondrodysplasia [107]. A number of BMPs like BMP-2, 4–6, 11 and growth differentiation factor 5 (GDF5) are reported to be secreted from both normal as well as OA adult human cartilage [108]. The role of locally secreted BMPs in cartilage biology inot yet clear, but a few studies have showed that BMPs play significant roles in protection and repair of cartilage by regulating the synthesis of aggrecan and proteoglycan [109,110]. Stages of chondrogenesis are followed by chondrocyte terminal differentiation and endochondral ossification. Apart from regulating the early process of chondreogensis, BMPs also participate during various stages of the chondrocyte terminal differentiation process [111]. During the formation of articular cartilage, terminal differentiation of chondrocytes is prevented, resulting in the permanent residence at the end of the long bones. However, during pathological conditions like OA, chondrocytes of articular cartilage undergo phenotypic changes, resembling terminally differentiating chondrocytes by secreting high levels of MMP-13 [112]. During differentiation, BMPs are required for hypertrophic state of chondrocytes along with the production of MMP-13. Participation of BMPs in terminal differentiation is proved by the fact that loss of Smad1 and 5 leads to blockage of chondrocyte terminal differentiation and severe cartilage defects [107]. Moreover, the overexpression of Smad6 or Smad ubiquitin regulatory factor 1 (Smurf1), inhibitor of Smad1/5/8 signaling cascade, in mice shows regular chondrocytes proliferation but blocked chondrocyte terminal differentiation [113]. Taken together, BMP signaling plays important roles not only in the early stages of chondryogenesis by stimulating the synthesis of matrix molecules like COL2, but can also participate during terminal differentiation by elevating MMP-13 expression, as observed in cartilages of OA [114].

BMPs are potent osteogenic stimulators and are able to regulate the activity of osteoblasts and osteoclasts *in vitro* and *in vivo* [115]. One recent study in *GDF5* haplo-insufficient mice demonstrated a decreased subchondral bone density and a distorted arrangement of collagen fibers in bone. It suggests that a reduced level of BMP signaling stimulator like GDF5 might affect the properties of subchondral bone [116]. Though there is not enough evidence for the role of BMP signaling in subchondral bone in joints, looking at the importance of BMPs in bone remodeling process, their participation during the progression of OA cannot be ruled out. Therefore, detailed studies focused on the role of BMPs in subchondral bone remodeling during OA are needed.

Along with BMPs, TGF-β also plays an indispensable role in articular maintenance, metabolic homeostasis and structural integrity [104]. TGF-β is expressed at high levels in normal cartilage while it is almost absent in OA cartilage [109]. Since, TGF-β is a potent inducer of cartilage ECM synthesis, interruption of TGF-β leads to loss of proteoglycans and cartilage degradation [117]. Moreover, ablation of endogenous TGF-β1 activity suppresses osteophyte formation and synovial thickening *in vivo* but exaggerates articular cartilage degeneration in osteoarthritis mice models [118,119]. Along with critical roles in cartilage homeostasis, TGF-β1 has been shown to regulate osteoclastic bone resorption and induces the migration of bone marrow mesenchymal stem cells (MSCs) to resorption pits for new trabecular bone formation in the long bones [120].

Evidence from discussed studies suggests TGF-β/BMP signaling as one of the prerequisites for normal homeostasis of cartilage-bone biochemical unit. An increased production of TGF-β from deteriorating cartilage under OA conditions [64] might affect homeostasis of both cartilage and bone in joints, pointing toward the possibility of an intermediate cross talk between cartilage and bone. Few studies in recent times have provided the proof of existence of this cross talk, affecting joint biology during the disease state. A direct effect of TGF-β from subchondral bone to cartilage was observed when an inhibition of TGF-β1 activity in subchondral bone attenuated its pathological changes and led to less degeneration of articular cartilage relative to untreated groups in different osteoarthritis rodent models. Subchondral bone of osteoarthritis mice model and OA human knee samples exhibited a high concentration of TGF-β compared to healthy controls. Increased TGF-β levels in subchondral bone induced OA like symptoms in mice: lower expression of proteoglycan; increased thickness of calcified cartilage; increase in volume fraction and number of blood vessels (angiogenesis) in subchondral plate; and higher number of MSCs in subchondral bone marrow undergoing osteogenesis to contribute *de novo* bone formation in subchondral bone. Inhibition of TGF-β1 restored the microarchitecture of subchondral bone by inhibiting angiogenesis and lowering the population of MSCs undergoing osteogenesis and also attenuates the loss of proteoglycan and increased calcification as observed in OA mice. As, subchondral bone and articular cartilage operate as a functional unit in joints, surgical induction of OA in mice led to thickness of subchondral plate and subsequent appearance of OA disease symptoms in cartilage, supporting the idea that changes in osteochondral bone is likely to affect the advancement of calcified cartilage zone during progression of OA [121].

Another possibility of cross talk between cartilage and subchondral bone comes from the secretion of various antagonists which could regulate the activity of BMP signaling during OA. A number of BMPs and their antagonists have been detected in normal and diseased cartilage [108,114]. BMPs and their antagonists might be necessary for maintaining normal homeostasis of cartilage and subchondral bone. But under pathological conditions, an unbalanced increase in expression may alter this delicate balance affecting either of the tissues leading to a disease state like OA. Indeed, increased expression of BMP signaling antagonists, gremlin, chordin, and follistatin has been reported in human OA chondrocytes compared to normal, and has been suggested to play regulatory roles during progression of OA [122]. Since BMPs are key regulatory molecules of the bone remodeling process, antagonists secreted at an elevated level from chondrocytes might be susceptible targets for intervention in subchondral bone treatment (Figure 2). However, to date, it is not clear to what extent locally secreted BMPs and their antagonists from cartilage may influence the BMP activity in normal and OA subchondral bone.

A crosstalk of WNT signaling pathway with TGF-β signaling has recently been reported. As discussed earlier, canonical WNT signaling activation can induce the secretion of WISP-1in human OA cartilages [101]. WISP-1 proteins are shown to possess osteogenic function by enhancing the differentiation process of osteoblasts [123]. Therefore, secretion of WISP-1 may have an osteogenic effect on underlying subchondral bone and alter its fate as observed in OA. Moreover, WISP-1 can modulate TGF-β signaling through inhibition of Smad2 [123]. Given the importance of TGF-β signaling in maintaining the homeostasis of cartilage and subchondral bone, interplay between TGF-β and WNT pathways by WISP-1 may play decisive role in determining the responses of cartilage and subchondral bone during OA.

Collectively, cross talk mediated by TGF-β/BMP signaling in either direction between cartilage and subchondral bone may provide new perspectives for understanding the pathophysiological processes of OA. Moreover, contribution of TGF-β/BMP signaling in the process of OA may further provide a rational framework for developing therapeutic interventions for its treatment.

#### 3.2.3. MAPK Signaling

Mitogen-activated protein kinases (MAPKs) are a set of serine/threonine kinases found in all eukaryotic organisms mediating a wide range of stimuli. The MAPKs family comprises three broad categories of kinases; extracellular signal-regulated kinases (ERKs), stress-activated protein kinases/c-Jun *N*-terminal kinases (JNKs) and p38 kinases. MAPKs have critical role to play both in cartilage and bone biology. Activation of all MAPKs has been detected in chondrocytes during chondrogenesis. In particular p38s and ERKs regulate the process of cartilage nodule formation during chondrogenesis, with p38 being necessary for the process, while ERK signaling represses it [124]. MAPKs are also likely to be involved in the transduction of mechanical signals in cartilage development [125]. In bone biology, the MAPKs family has been demonstrated to play essential roles in the regulation of bone mass by controlling the process of osteoblast and osteoclast differentiation. ERK signaling mediates both early and late differentiation processes of osteoblasts by phosphorylation of key transcriptional factors like RUNX2 and activating transcription factor 4 (ATF4). Similarly, p38 signaling exerts its effect to promote osteoblast differentiation through phosphorylation of DLX5, Osterix and at least in parts through RUNX2. ERK, p38, and JNK all appear to promote osteoclast differentiation by regulating activator protein 1 (AP-1) as a critical mediator of osteoclastogenesis. Moreover, bone cells respond to extracellular matrix binding and mechanical loading through ERK activation [126]. Of late, the MAPKs family has been attributed to be involved with the pathophysiology of OA. ERK and p38 activation are key upstream signaling events in processes leading to degeneration of articular cartilage. Activation of both, ERK and p38 signaling are essential for MMP expression and activity, while only ERK activation is essential for aggrecanase-mediated cartilage degradation [127]. Mechanical strain induction in osteoblasts also results into MMP-13 production through ERK activation in osteoblasts [128]. Initiation of MMP-13 production from osteoblasts of subchondral bone in response to mechanical strain may instigate the degradation of cartilage as observed during progression of OA. The possibility of MAPKs-mediated release of degradative enzymes from subchondral bone affecting chondrocytes suggests the existence of intercellular communication between cartilage and bone affecting each other. Co-culture of normal or OA subchondral bone with articular cartilage and *vice versa* elucidated an intercellular cross talk between both tissues mediated by MAPKs [128,129]. OA articular chondrocytes enhanced osteoblast differentiation markers such as RUNX2, alkaline phosphatase, osteopontin and osteocalcin in normal subchondral osteoblasts, while normal articular chondrocytes appeared to delay the process of differentiation. Changes induced by OA articular chondrocytes to normal subchondral osteoblasts seemed to be mediated by ERK phosphorylation in osteoblasts [128]. Another study demonstrated that OA subchondral osteoblasts stimulate hypertrophic gene expression and matrix calcification in articular chondrocytes by inducing ERK signaling activity and down regulating p38 signaling activity in co-cultured articular chondrocytes. This effect was observed both in direct chondrocyte pellet culture on osteoblast monolayer cultures and in chondrocyte pellet culture grown in medium conditioned by OA subchondral osteoblasts [130]. Moreover, indirect co-culture of normal articular chondrocytes with medium conditioned by OA subchondral osteoblasts aggravated the proteolytic activity and increased expression of ADAMTs5, ADAMTs4, MMP-2, MMP-3, and MMP-9 in normal articular chondrocytes. While culturing of normal subchondral osteoblasts with medium conditioned by OA articular chondrocytes caused an increase in MMP-1 and MMP-2 expression in normal subchondral osteoblasts. Upregulation of ADAMTs and MMPs in the both cases was mediated by the activation of the ERK signaling pathway in respective cells. Inhibition of ERK signaling pathways with inhibitor PD98059, both in affected articular chondrocytes and subchondral osteoblasts reversed the overexpression of ADAMTs and MMPs in co-culture systems. Secretion of aberrant amounts of ADAMTs and MMPs in OA joints, play crucial roles in the early stages of OA development. Results obtained from this study suggests that altered bidirectional signaling between subchondral osteoblasts and articular chondrocytes significantly influences the critical features of both cartilage and bone by producing abnormal levels of ADAMTs and MMPs. In addition, unknown soluble factors released from OA articular cartilage or subchondral bone mediates release of ADAMTs and MMPs through activation of ERK signaling in normal subchondral bone or articular cartilage, respectively [131]. Further studies are needed to identify these secreted soluble factors to understand intercellular communication between articular cartilage and subchondral bone for therapeutic interventions. These *in vitro* studies clearly implicate MAPKs as mediators of cross talk between articular cartilage and subchondral bone but further studies are warranted to validate these findings at *in vivo* and clinical level.

Proteinase-activated receptors (PAR-2) are a novel family of seven-transmembrane G-protein-coupled receptors. Instead of activation by circulating agonists, they are activated enzymatically through proteolysis of the receptor. This proteolytic cleavage is specifically mediated by the serine proteases [132]. Both osteoblast and chondrocytes expresses membrane bound PAR-2 on their surfaces. OA articular cartilage and subchondral bone produces significantly increased level of PAR-2 compared to normal [133,134].

Presence of proinflammatory cytokines IL1β, TNFα and growth factor TGF-β can upregulate the expression of PAR-2 in cartilage explants. PAR-2 activation upregulates the expression of important catabolic and proinflammatory mediators like MMP-1, MMP-13 and COX-2, involved in the progression of the disease and the effect is mediated by the ERK and p38 signaling activity in OA cartilage [133]. Similar upregulated expression of PAR-2 was also observed in subchondral osteoblasts in the presence of proinflammatory cytokines IL1β, TNFα and inflammatory mediator prostaglandin E-2. Activation of PAR-2 in OA subchondral osteoblasts plays a role in subchondral bone resorption by increasing the expression of MMP-1, MMP-9, RANKL and IL-6 but not osteoprotegerin or MMP-13. The PAR-2 effect is mediated by activation of ERK and JNK but not by p38 signaling in osteoblasts [134]. Protection against cartilage damage and bony changes in PAR-2 knockout mice on induction of a meniscus destabilization OA model further corroborates the participatory role of PAR-2 in pathophysiology of OA. The reported presence of inflammatory cytokines from cartilage of OA patients may mediate a cross talk between cartilage and subchondral bone in terms of modulation of PAR-2 expression. Involvement of PAR-2 as a stimulator of MAPKs in inducing OA-like symptoms provides an attractive target for the treatment of OA, as controlling the expression of PAR-2 may not only reduce OA symptoms but may also likely slow down disease progression.

## 4. Conclusions

Both cartilage and subchondral bone form a biocomposite functional unit that is uniquely adapted to the transfer of loads across the diarthrodial joints. Close proximity of cartilage and subchondral bone provides an ample opportunity to induce physical and functional alteration in each other through molecular interaction. Recent studies reporting the presence of blood vessel innervations from subchondral bone into cartilage zone, and the existence of microcracks and fissures both in cartilage and subchondral bone support the view of transfer of molecules between the two tissues. Several biological factors and signaling molecules produced from both tissues may passage from one zone to another, affecting homeostasis of neighboring tissue. Current studies both at *in vitro* and *in vivo* levels provide meaningful evidence of cross talk between cartilage and subchondral bone in synovial joints. Secreted cytokines, growth factors and signaling molecules form cartilage-bone biochemical units can play modulatory roles to alter pathophysiology of joints during OA. Given the importance of WNT, BMP, TGF-β and MAPK signaling in controlling joint homeostasis, subsequent maintenance and their role in cross talk between cartilage and subchondral bone make them exciting candidates for understanding the various parameters that results into alteration of normal joint homeostasis. Moreover, interplay between signaling pathways like WNT, BMP, TGF-β and MAPK in both cartilage and subchondral bone might represent a more complex network of interaction than expected, and thus needs a cautious approach to delineate their role in progression of OA.

Questions still persist about the contribution of other factors like cytokines and growth factors released from joint tissues other than cartilage and subchondral bone in molecular interactions that exists in OA joints. The eventual goal in treatment of OA could be achieved either by recovering or protecting the joints undergoing destruction. Increasing understanding of the molecular interaction between the cartilage and bone in joints during OA will open up new avenues for treatment. Particularly, modulation of signaling mechanisms mediating deleterious effects of cartilage and subchondral bone and *vice versa* would be promising for future therapeutics. In conclusion, multidisciplinary approaches and extensive molecular studies focused on interactions between cartilage and bone can provide new insight for understanding the pathophysiology of OA as a multifactorial disease and improving existing therapeutic strategies.

## Figures and Tables

**Table 1:** Biomarkers of cartilage and subchondral bone during onset and progression of Osteoarthritis (OA).

Biomarkers	Function in joint	During OA elevated expression represents	References
*Biomarkers for Cartilage*			
Cartilage oligomeric matrix protein (COMP)	Help in inflammatory proliferation of synovial membrane, regulation of fibril assembly and to maintain the mature collagen network	Cartilage degradation	[28]
*C*-terminal telopeptide of type II collagen (CTX-II)	provide strength, integrity and maintain shape of tissue	Remodeling of calcified cartilage	[29]
Helical fragments (Helix-II and Coll 2-1, Coll 2-1 NO_2_)	Contribute in inflammatory processes and cartilage catabolism in the joint	Type II collagen degradation	[30]
Amino-terminal type II procollagen propeptide (PIINP)	One of the two propeptides of type II procollagen and rflect the rates of collagen type II synthesis	Cartilage degradation	[31]
Carboxy-terminal type II procollagen propeptide (PIICP)	One of the two propeptides of type II procollagen and reflect the rates of collagen type II synthesis	Cartilage degradation	[31]
YLK-40 Glycoprotein: noncollagenous protein	Have a vital role in creating or amending tissue inflammation, immunity and/or remodeling	Cartilage degradation	[32]
Keratan sulfate (KS-5D4)	Act as a cushion to absorb mechanical shock.	Aggrecan and cartilage degradation	[33]
Chondroitin sulfate 846 epitope (CS 846)	provides a hydrated gel structure (via its interaction with hyaluronan and link protein) that endows the cartilage with load-bearing properties	Cartilage turnover	[28]
Hyaluronic acid (HA)	Essential for viscoelasticity of synovium fluid and cartilage	Cartilage degradation	[34]
*Biomarkers for Bone*			
*N*-terminal type I collagen telopeptides (NTX I)	Maintain bone remodeling process	Type I collagen degradation	[35]
*C*-terminal type I collagen (CTX I) or Serum CTX I	Crosslinking peptide of type I collagen and necessary for immunoreactivity	Increased osteoclastogenesis and Bone degradation	[35]
Amino-terminal procollagen propeptide of type I collagen (PINP)	One of the two propeptides of type I procollagen and represent synthesis rate of type I collagen	Bone degradation	[36]
Carboxy-terminal procollagen propeptide of type I collagen (PICP)	One of the two propeptides of type I procollagen and represent synthesis rate of type I collagen	Bone degradation	[36]
Osteocalcin (OC)	Essential for bone mineralization and recruitment of osteoblast and osteoclast at the site of bone formation	Anabolic bone turnover	[28]
Urinary total pyridinoline (PYD)	Contributes to stabilizing and reinforcing the whole structure of collagenous tissues as bone and cartilage	Catabolic bone turnover	[28,37]
Bone sialoprotein (BSP)	Necessary for mineralization at cartilage bone interface	Anabolic bone turnover	[37]
